# Supplementary figures and images for: Abundant genetic variation is retained in many laboratory schistosome populations
Source: PLoS Pathog. 2025 Aug 20;21(8):e1013439. doi: 10.1371/journal.ppat.1013439 (PMC12377630; doi:10.1371/journal.ppat.1013439)

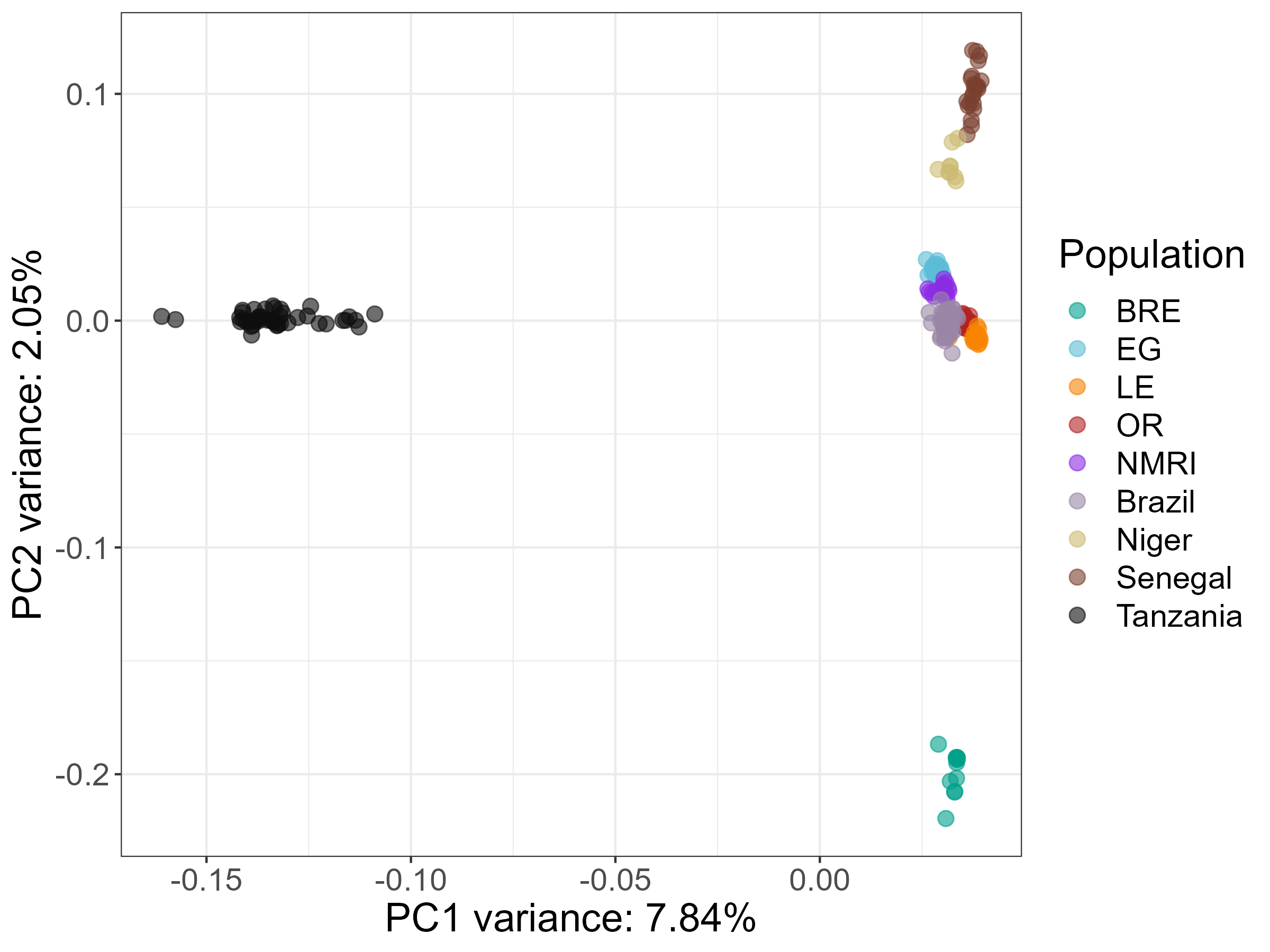

Supplement: S1 Fig — PCA plot showing clusters of all populations used in this study. (TIFF) [file ppat.1013439.s001.tiff]

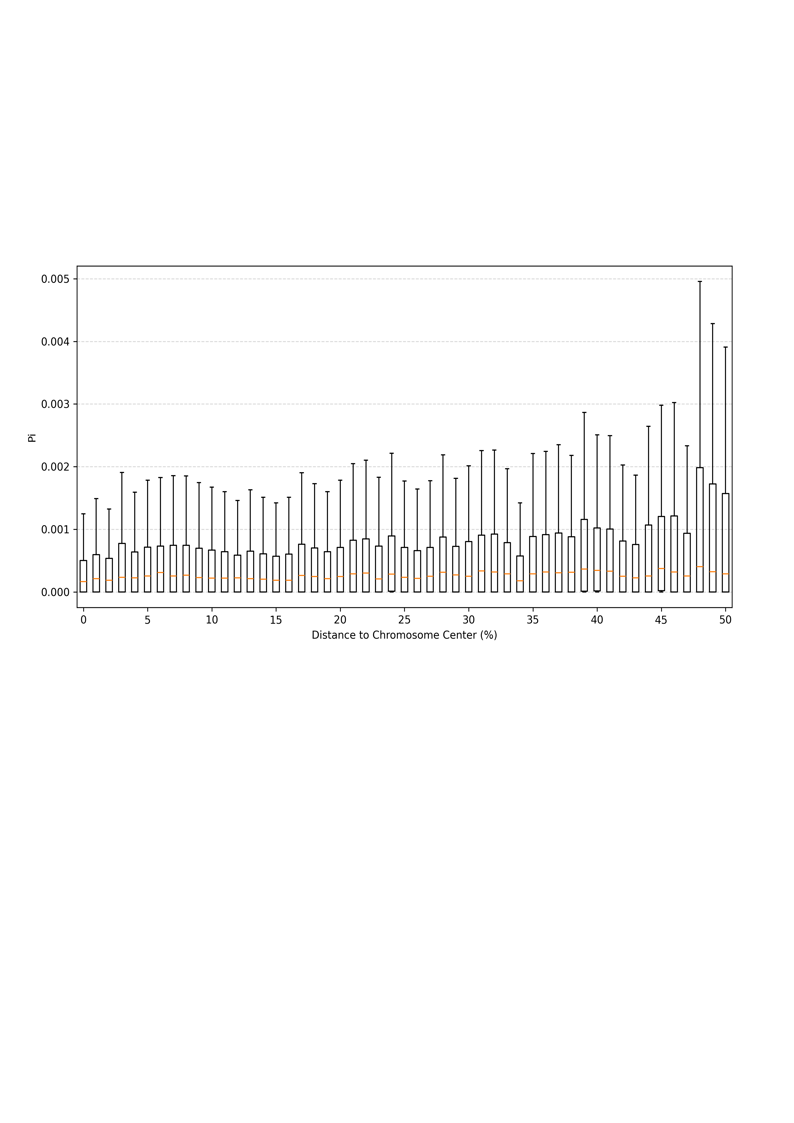

Supplement: S2 Fig — We calculated nucleotide diversity (π) in 25kb windows separately in each laboratory S. mansoni population. The distance from each window to the nearest chromosome end was calculated as a percentage of the total chromosome length and binned to the nearest whole percentage point. Box plots indicate the range of π values across all populations at each particular distance bin. There was no relationship between mean π and proximity to the chromosome ends, but variance in π increased at the chromosome ends. This result was also obtained with windows of 5 and 2 kb, so is robust to window size. (PNG) [file ppat.1013439.s002.png]

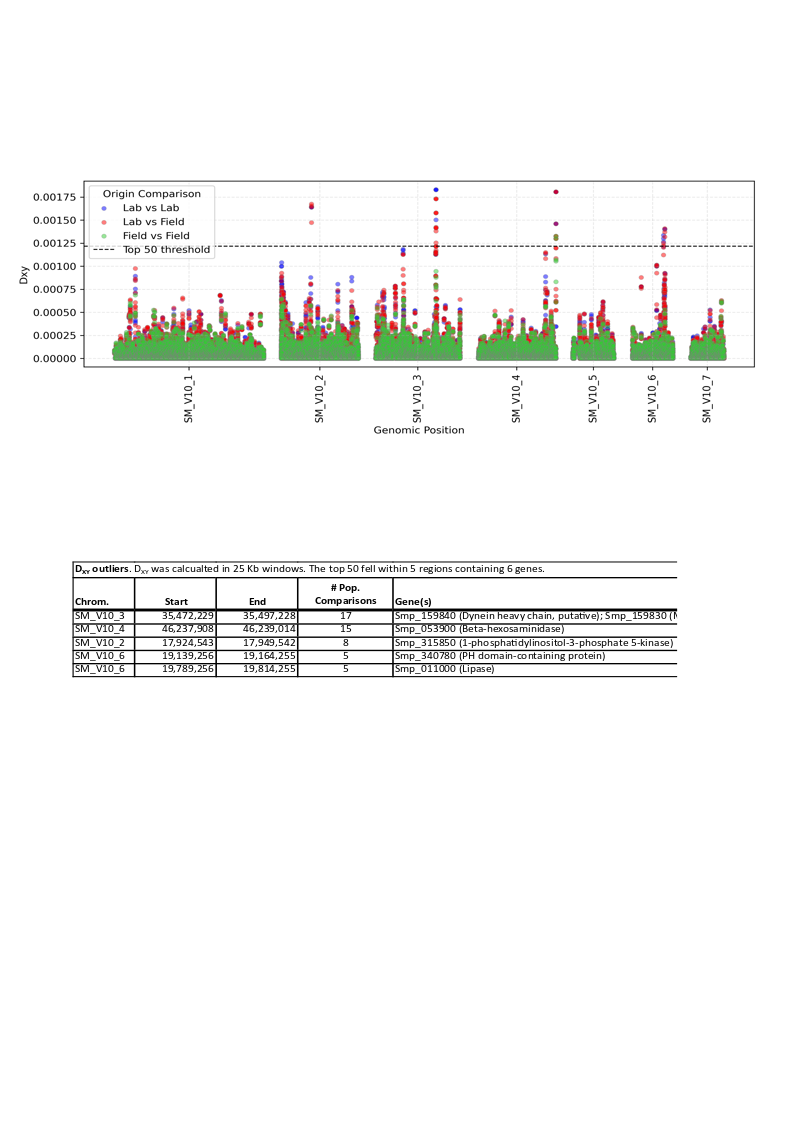

Supplement: S3 Fig — Data from all pairwise combinations are plotted on the same graph: red dots indicate comparisons between lab and field populations, green dots field vs field populations and blue dots are lab vs lab populations. The gene content in the DXY peaks are shown in the table below. (PNG) [file ppat.1013439.s003.png]

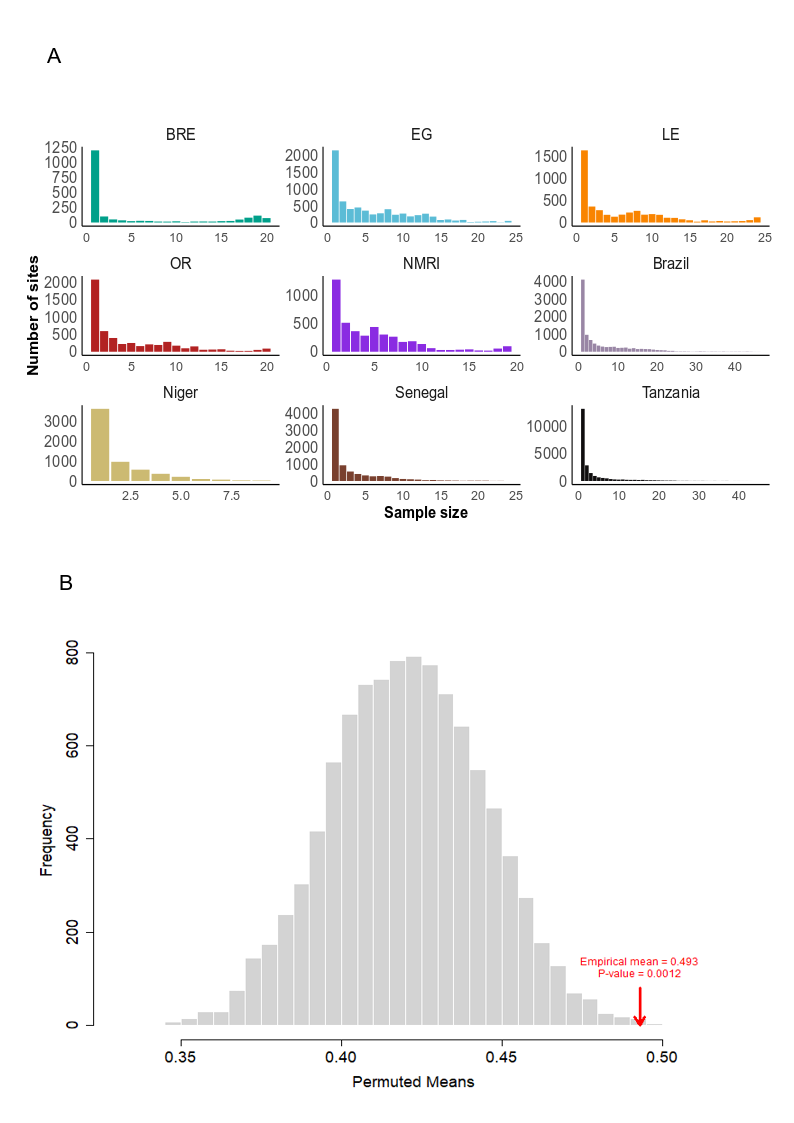

Supplement: S4 Fig — (A) Histograms of folded allele frequency spectra of each S. mansoni population. (B) Permutation tests to compare ECDFs from field and laboratory populations. We conducted pairwise comparisons between all populations examined (36 pairwise comparisons). We then calculated the mean K-S statistic for 16 comparisons of field and lab populations, and compared this to 10,000 randomly permuted datasets. The histogram shows the distribution of permutated values, with the empirical value and one tailed permutation test statistic marked by the red arrow. (PNG) [file ppat.1013439.s004.png]

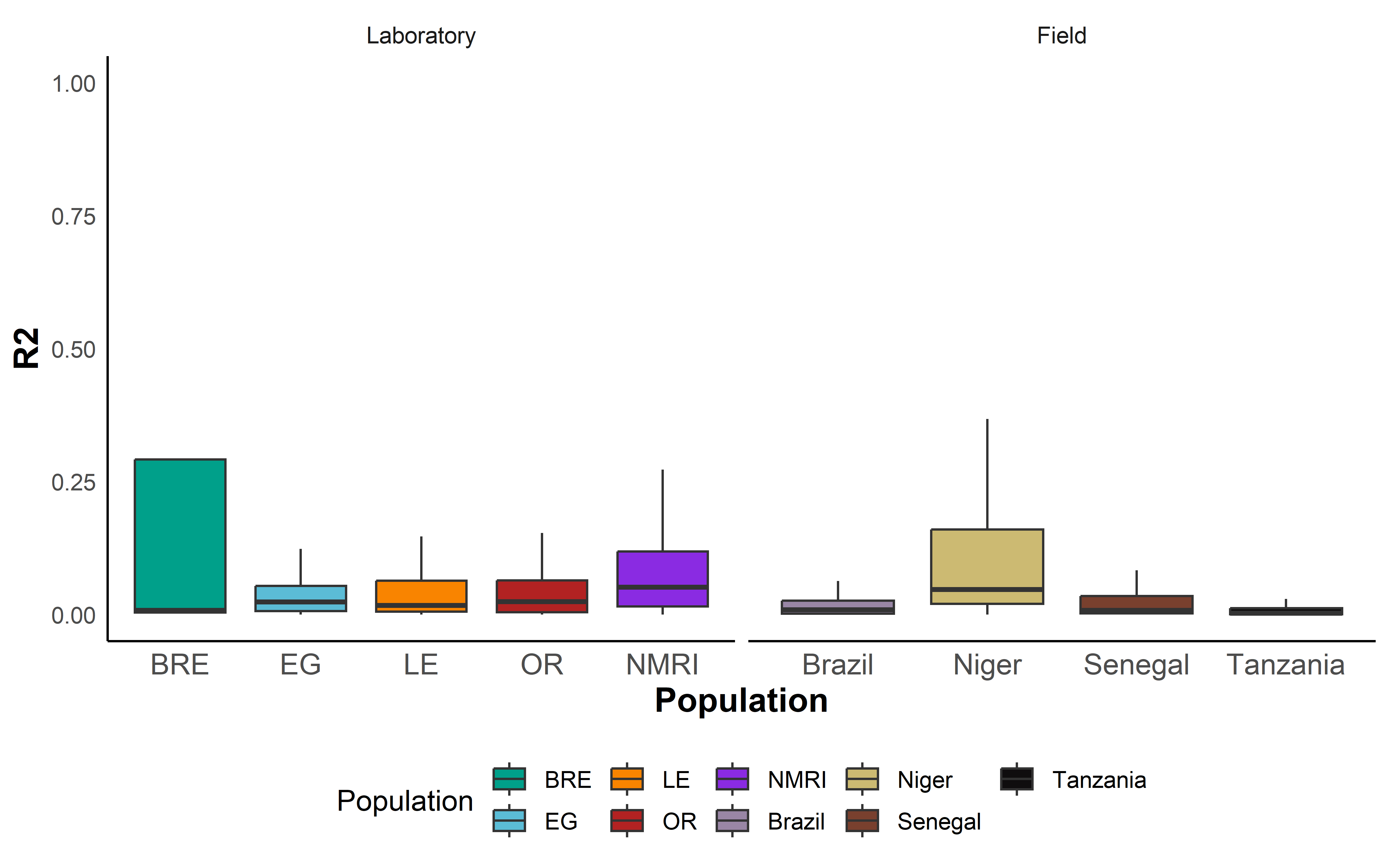

Supplement: S6 Fig — Box and whisker plot showing LD (squared correlation coefficient, R2) of unlinked variants in each population. (TIFF) [file ppat.1013439.s006.tiff]

**A**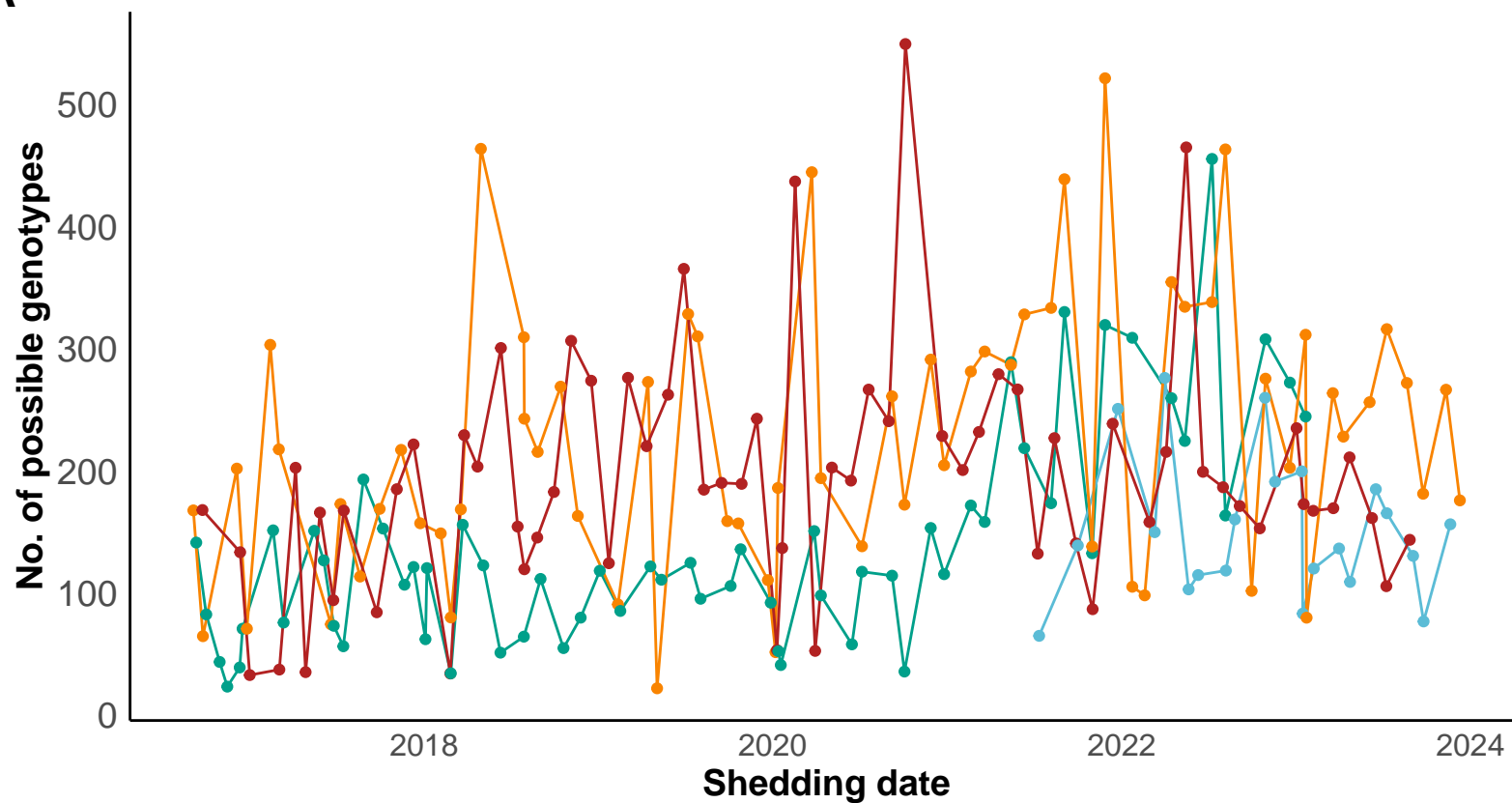

Population — BRE — EG — LE — OR

**B**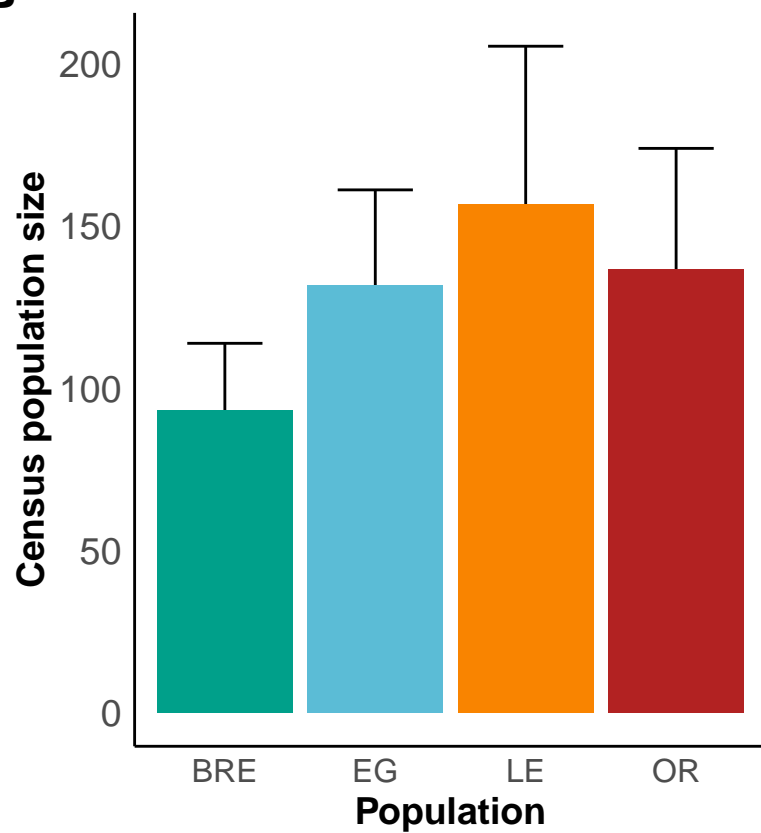**C**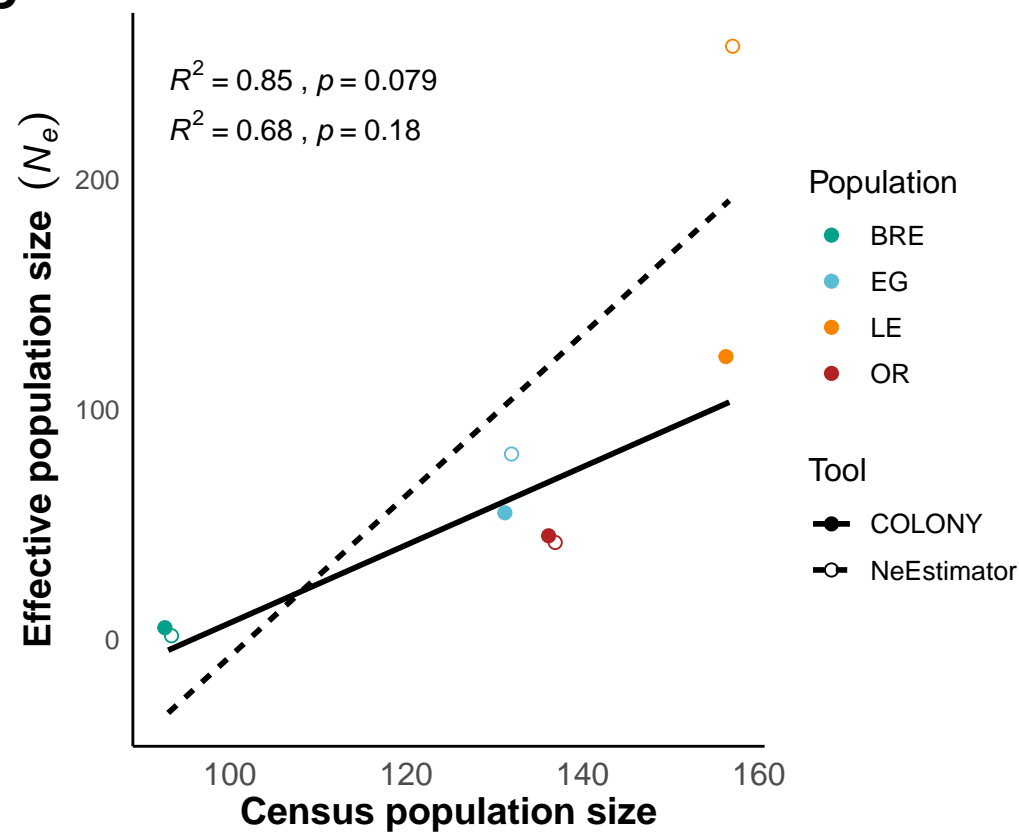

Supplement: S7 Fig — (A) Line plot showing estimated census size over time. We used detailed life cycle maintenance records to estimate P(0) and calculated numbers of parasites/snail assuming a Poisson distribution. Note that these Nc values are likely to be systematic overestimates. We conduct hamster infections with newly infected batches of snails to which we add surviving infected snails from the prior life cycle maintenance. Therefore, the proportion of uninfected snails (P(0)) will be underestimated, and Poisson estimates of numbers of parasite genotypes per snail will be overestimated. The actual Nc values are likely to be somewhat lower. (B) Bar plot showing the harmonic mean of the Nc for each population. The error bars represent a 95% confidence interval. (C) Scatter plot showing the relationship between Ne as calculated by COLONY (filled circle) and NeEstimator (open circle) for each population. The lines represent a linear regression model, and the corresponding Pearson correlation coefficients are displayed in accordance with the legend of the tool used. (PDF) [file ppat.1013439.s007.pdf]
