# Supplementary material for: Abundant genetic variation is retained in many laboratory schistosome populations
Source: PLoS Pathog. 2025 Aug 20;21(8):e1013439. doi: 10.1371/journal.ppat.1013439 (PMC12377630; doi:10.1371/journal.ppat.1013439)
Supplement: Attachment — Submitted filename: PLoS Pathogens D 24 02401.docx [file ppat.1013439.s010.docx]

**PLoS Pathogens D 24 02401**

This is an interesting ms. that describes genome sequence analysis of lab lines of the trematode *Schistosoma mansoni*, and compares them to already available genome data for wild parasites. The authors show that the lab lines, as one might expect, had less genetic variation than the wild parasites, though that the lab lines were not fully inbred. The authors discuss the utility of the lab lines in schistosome research, and present caveats to this. Overall the work is thorough, and the conclusions are justified by the data that are presented.

I think that the ms. could do quite a bit better in reaching out to a wider readership. As it stands it is readily comprehensible to those working on trematodes, but some matters wouldn’t be clear to other parasitologists, and certainly not to other pathogen biologists. I offer some suggestions in my more detailed comments below.

Throughout, some greater care and precision is warranted, again which I also lay out below.

I’m unclear about the 19 to 24 parasites sequenced from each laboratory line (line 23; lines 119 – 121; the methods; Figure 2 and its legend). Methodologically (from line 247) was each snail infected with a single miracidium? If so, for infected snails, was it a pool (size 19-24) of the cercariae from each infected snail that was then sequenced? (As an aside, in Figure 2’s top panel is the beaker meant to show eggs in water that are hatching to release miracidia?) If it is pools of cercariae from single-miracidia-infected snails that were sequenced, isn’t this effectively a severe bottlenecking, and does this lead to the low estimates of genetic variation present with each lab line? To put it another way, if offspring of different parasites within each laboratory line were sequenced, would the estimates of genetic variation be higher? Alternatively, it is cercarial pools of single-miracidia-infected snails that were harvested, and then 19-24 of these pools per lab line that were sequenced? As it stands, the text and figure isn’t sufficiently clear to understand this rather important point.

I am surprised by the admixture results (lines 137-139) given that the lab lines are maintained with sexual reproduction and that there are decent levels of genetic variation within the lines. It makes sense that there is no mating among the lab lines (though line 371 talks about cross-lab line contamination and, I assume, crossing), but have the authors examined the admixture of each lab line separately?

To my taste the Discussion is overly long and digressive, though I think this about almost all papers I review, and of many papers I read – so this might say something about my taste.

**Detailed comments**

Line is 20 to 21. “Schistosomes” and “rodents” – can all schistosome be maintained in rodents? I presume this hasn’t been tested for all species.

Line 29. “Indicative” – this is ambiguous because given the text before this word it could refer to “all laboratory populations” or “SmBRE”.

Line 44. This “clonal” doesn’t make sense to me here. This is also repeated on line 67.

Lines 48 – 50. This sentence is an opportunity for the authors to give a broader perspective about *S. mansoni*, for example telling the wider leadership that this is a macroparasite, a trematode, a platyhelminth *etc*.

Line 51. I don’t think that the authors can conclude that the genetic variation was ”reduced”, because they don’t present data showing the genetic variation in the ancestors of these lab parasites. Rather the authors can only say that the variation currently present in the lab lines is approximately half of that present in present day wild populations. This is repeated on line 113, line144 (here how about “significantly lower” rather than “significant reduction”?), and line 212.

Line 64. What is meant by “recombining”? Do the authors mean meiotic recombination or do they mean quasi-random sexual meeting among adult parasites, or something else?

Line 65. The sentence beginning “successful” seems quite out of context and I suggest that it is removed or revised by the authors. As toa revision, I think the text needs to explain the reason for telling the reader this.

Line 66. Sentence starting “Schistosomes”, could be an opportunity to explain the life cycle and particularly the genetics of the life-cycle to a wider readership. As it currently stands this sentence doesn’t make the important point that the adult parasites undergo sexual reproduction. I’ve commented above about the use of the word “clonal” and wonder if “asexual” may be better here.

Line 76. Are these five generations sexual generations or asexual generations?

Line 93. How about “difficulties” or “imperfect genome assemblies” rather than the (overused and often largely meaningless) “issues”?

Line 95. Insert “assembly” after “genome” – the *S .mansoni* genome is perfect, it is just our knowledge and our assembly of it that is imperfect.

Line 96. Another “issue”, but it isn’t clear what “this” refers to.

Line 97. This would be a place where the authors could give some brief details about the genome assembly, which otherwise is barely mentioned or metrics about it given. Also, while reading this ms. I realise I have no idea of the size of the *S. mansoni* genome assembly.

Line 99. “Cercariae” will be almost meaningless to non-schistosome biologists and certainly to non-parasitologists, and so this is another example of where the authors could reach out to a wider audience.

Line 105. How about “the present study” rather than “this study”?

Line 108. Figure 1 doesn’t obviously support the two alternatives presented in the text here.

Line 119 and Figure 2. I am unclear what the 19-24 parasites per lab line are, as I explain above.

Lines 124 –125. What are the n’s for each of the countries?

Table 1. What is a “sample” in the heading of the second column. And, instead of “All variants” how about “Total variants”?

Lines 134 to 136. This is wrong in two ways. First, the four lines are separated by the vertical access, they are clustered by the horizontal axis. Two, they are separated / clustered by the principal components not the axis *per se* – a pedantic point but principal components 1 and 2 could equally well be plotted on the y and x axis, respectively.

Line 153. I don’t know what this Wilcoxon test is testing and I can’t see that this is explained in the methods.

Line 159. I don’t understand what this statistic is telling us about the ECDF – is it an analysis of the slope or the intercept, or something else? I can’t see that this is explained in the methods. Also the P value here is different than that on Figure 5B, but I think that they should be the same.

Line 188. Why the harmonic mean?

Line 189. How was this number of genotypes estimated? Line 451 *et seq* of the Methods seems to use the number of miracidia but these aren’t the definitive host infective stage and how do the authors get from miracidia number to genotype number?

Table 3. Six of these columns repeat data in Figure 7. There is no legend to the Table.

Line 198. Is “N” the number of worms, and if so the number of which life cycle stage?

Line 239. Sentence beginning “We observed…” doesn’t sit easily with me. Didn’t the authors observe a positive Tajima’s D, which is because of the comparative loss of rare alleles. In itself, the authors didn’t observe a loss of rare alleles (thought there is Figure 5B, though there are no analyses of these date). [I downloaded what purported to be Figure S1 (9 histograms with the axes labelled ‘Number of sites” and “Sample size’, but this isn’t obviously what line 156 refers to.] Notwithstanding, my comment concerns the logic of what is observed and what is inferred, which I’m not sure is correct in this sentence.

Line 241. Insert “comparative” before “deficit”?

Line 247. Why “reduced … recombination events”?; and see my comment on line 64.

Line 262. What are the “groups”?

Line 264. The sentence beginning “A microsatellite” seems out of context. Explain the reason for telling the reader this.

Line 299. ExamineS.

Line 306. I wonder whether “association” is helpful here since a GWAS seeks statistical associations between the phenotypic trait and genetic markers, but GWAS works because genetically there is linkage (another type of association) between a locus underlying trait and genetic markers.

Line 347. “so are typically…” was this exclusion done here? If so, I didn’t get that from reading the methods.

Line 374. Perhaps clarify that the heads were what was sequenced.

Line 464. How were the 95% confidence intervals calculated?

Figure 2A. Why are the sex chromosomes not shown?

Figure 6A. This plot shows dots through which lines are drawn. What are the dots and what are the lines? The Methods doesn’t obviously explain this.

Figure 8. The y-axis label is unclear to me, and I can’t see that “H0” is explained in the Figure legend or the text,
