# Supplementary material for: Abundant genetic variation is retained in many laboratory schistosome populations
Source: PLoS Pathog. 2025 Aug 20;21(8):e1013439. doi: 10.1371/journal.ppat.1013439 (PMC12377630; doi:10.1371/journal.ppat.1013439)
Supplement: Attachment — Submitted filename: 2024_PLoS_Pathogens_Schisto_lab_strain_diversity.docx [file ppat.1013439.s011.docx]

2024_PLoS_Pathogens_Schisto_lab_strain_diversity

Jutzeler and colleagues have analysed the genetic diversity of five laboratory-adapted populations of *Schistosoma mansoni* using whole-genome sequencing. Surprisingly, they found that, despite being isolated for decades and hundreds of generations, the laboratory-adapted isolates were still relatively genetically diverse compared to field isolates. These data have important implications for understanding genetic and phenotypic variation in laboratory models, which are usually considered to not be diverse. The discussion was particularly good in highlighting the strengths and limitations of having such diversity in lab populations and the fact that it might be exploited to understand diversity and trait variation better, especially if single-worm phenotyping is used. Similarly, extending this to other helminths is important, although there are more examples that could have been drawn from.

Overall, the manuscript is well written, its analyses robust, and its conclusions are generally well supported. Below are some questions and comments that I think are worth considering. Addressing them may strengthen the manuscript and clarify potential uncertainties for the reader.

Kind regards,

Stephen Doyle

Wellcome Sanger Institute

General comment

- An overall theme is comparing lab to field strain diversity; however, one aspect of this analysis is the data have not been generated in the same way, i.e., whole genome sequencing was used for the lab stains, and exome sequence was used for the field strains. Filtering variants to include only coding variants helps bring these two approaches together. However, are there other sources of variation that need to be accounted for? For example, how concordant are WGS and exome capture variants? Are there coding sequences that the exome capture does not target, and do you account for this here? Is there any allelic imbalance caused by the exome capture that might not be affected by WGS? This technical variation could use some recognition in the discussion.
- Reading throughout, I keep returning to the statements of “49%... diversity retained” and “51% reduction.” You have multiple independent populations of both field and strain, within which there is distinct variation within each, so it doesn't make sense to me to make a singular statement like this. “Retained” and “reduction” suggested you measured diversity in the starting population, time passed, and then measured again, i.e., one population changed over time. However, with independent populations, this statement can’t be made, at least not precisely, as there is no defined history or genetic relationship between field and lab strains. Moreover, some of the variations in the lab populations will be new since they were isolated and, therefore, distinct, i.e., different between the same lab isolates kept in different labs, for example. I think this deserves some thought as to how you describe it. Perhaps framing it as the “relative levels of genetic diversity” might be suitable, and simply that there is more variation in lab strains than expected.
- I don't think Figure 1 or Figure 2 are meaningful or detailed enough to warrant being in the main text figures.

Specific comments

Line 96: “This approach contributed to a much-improved genome “

- This seems to end prematurely, as there is an even more contiguous genome assembly (*S. mansoni* V10 in WBPS; Buddenborg et al bioRxiv) that was also improved with DNA derived from single miracidium infections.

Line 123: “Detailed information about these variants is listed in Table 1.”

- Given the sample sizes, the minor allele frequency filter may or may not retain SNPs present only in a single individual. E.g. Niger will be very susceptible to this with only nine individuals.
- How meaningful are these rare variants, and are they found in multiple populations to give some confidence that they are real?
- Did you look at the number of SNPs fixed in different populations?

Line 132: “Principal component analysis (PCA) and admixture”

- This is clear that the laboratory populations are distinct, which is great. Perhaps not in the same way, though; for example, there is minimal variation in PC1 separating four strains. Do you have any idea what is driving the divergence on PC1?
- However, it seems like a missed opportunity that you have not compared them to the field populations. I suggest that if not here, then in the supplement, you should show all of the samples in the PCA.
- Given you have both a lab and field strain from Brazil, it would be interesting to know how similar they were.

Line 141: “Nucleotide diversity in S. mansoni laboratory and field populations”

- It is interesting that SNP variation is relatively consistent across the genome and that it is not just restricted to chromosome ends, which is commonly seen. It might suggest there are technical aspects of variant calling in these regions or true hotspots
- It would be helpful to show some pairwise comparisons of nucleotide diversity using Dxy or something similar. Even a ratio of Pi would work. It would help highlight population-specific regions of high diversity.

Line 143: “Statistical analysis using a Kolmogorov-Smirnov test showed a significant reduction in diversity (51%) in laboratory populations (D = 0.403, p < 0.001).”

- This is a little confusing, as it is unclear what is being compared from the figure.
- First, the KS test compares whether two datasets are drawn from the same distribution—so are you comparing all fields against all labs as two groups? If yes, is that appropriate?
- The 51% reduction is based on what exactly? Cumulative diversity in lab vs field? The figure doesn't show this, given that you have shown each population.

Line 153: “Wilcoxon test”

- What are you testing here, exactly?
- The test is not significant; however, your description of the test does not reveal that. For example, you say that “all field populations showed negative values ” with a non-significant test. Does that mean that statement is not supported? It's best to clarify.

Line 158: “significant differences between allele frequency spectra for laboratory and field populations”

- Again, it needs to be clarified what the KS test is comparing.

Line 166: “To compare LD decay curves, we measured the distance at which LD is reduced to r2 = 0.5 (LD0.5, Figure 6B).”

- The problem with this is none of the populations get to 1, and that Tanzania doesn't get to 0.5, so it has no value.
- It would be better to calculate the LD at 50% of the max LD per population
- It should also take into account the background LD, which could be determined by the LD between variants from different chromosomes.

Line 167: “Figure 6B”

- This is the most telling difference between lab and field populations, and given the WGS vs. exome confounder, the difference is likely underestimated.

Line 169: “LD decay was nearly significantly”

- It can't be nearly significant. It either is, or it isn't. Here, it is not significant. Please change.

Line 196: “Simulations of genomic diversity in populations of different size”

- This is neat.
- How did you account for the lifecycle in your simulation? The methods are not very clear and do not account for an intermediate host or clonal stage in the life cycle.
- If I interpret this correctly, it suggests that with a comparable effective population size and generation time, one might not expect to lose much diversity. Is that interpretation correct? If so, the overall result of “retaining” diversity is not surprising.
- How dependent is this result of maintaining diversity on the actual sample in which diversity is maintained? Could you get this result independently (including considering the life cycle)?

Line 216: (Table 4)

- I’m not convinced a table like this enables a meaningful comparison between studies or organisms. There is so much variation between all of these examples - time, population size, bottlenecks, life cycles etc - and presenting a table with individual figures and a reduction in diversity almost implies this is a biological truth to these numbers rather than a random collection of anecdotal evidence.
- Nonetheless, I am more concerned that you have simplified your data into a single data points, when clearly the rest of your data shows variation between samples and between groups.
- I suggest you remove the table. It is OK to reinforce that “domestication” or “lab adaption” generally results in loss of diversity. Still, I think it is best not to make comparisons when they can’t properly be compared.

Line 221: “laboratory schistosome populations are typically founded by collecting eggs from one or more patients, each of which may be infected with hundreds of adult worms”

- This is hypothetical—I appreciate we cannot know exactly—but let's assume this is true. Given your effective population size estimates, it would potentially suggest there is effectively no loss of diversity from a small founder population over hundreds of generations. Does this make sense?
- I think this needs some more thought and discussion. Comparison to the field populations is actually too broad, and would be more informative to compare to the founder population.

Line 332: Relevance to other helminths

- This is a really important point to raise, and might prompt similar studies to better understand variation in other laboratory models.
- However, you briefly describe only one helminth and C. elegans - what about the others? There are a few I can think of, eg. Brugia malayi (Mattick et al 2021 PLoS NTD).
- Given you have made a point to illustrate it, I think it is worth fleshing this out a little more.

Line 414: “we lifted the file over to v10 of the S. mansoni reference genome (Wellcome Sanger Institute, project PRJEA36577) using LiftoverVcf.”

- How accurate is this? What validation did you do?
- Why did you not simply remap and call variants again?
- To what degree might this influence your results?

Line 420: “and > 56% missingness,”

- This is an interesting number - how did you come up with it?
- There is also a difference in the filtering strategy between the lab and field samples - what effect might this have on the comparison?
