# Supplementary material for: Abundant genetic variation is retained in many laboratory schistosome populations
Source: PLoS Pathog. 2025 Aug 20;21(8):e1013439. doi: 10.1371/journal.ppat.1013439 (PMC12377630; doi:10.1371/journal.ppat.1013439)
Supplement: Attachment — Submitted filename: Response to reviewers.docx [file ppat.1013439.s013.docx]

We thank the reviewers for their constructive comments and suggestions. Changes to the revised manuscript are shown in the “changes marked” version. In brief, we have made several key modifications suggested in the revised manuscript:

- We have now reanalyzed the sequence data using filtering parameters that allow more robust comparison between laboratory (whole genome sequence) and field collected parasites (exome capture) sequences. We then used the new filtered dataset to redo the comparative analysis of field and lab populations.
- We have removed (Figures 1 and 2) as suggested.
- We have added PCA plots comparing all population (both lab and field) to the supplement
- We have added new analyses (suggested by Reviewer 2) to compare levels of fixed derived SNPs in each population, to examine population specific regions of high diversity (D_XY_), and to evaluate patterns of diversity across the chromosomes
- We have examined LD between unlinked SNPs on different chromosomes to evaluate background LD.
- We have replaced some overly simplistic statistical analyses in the initial draft, with more robust statistical methods.
- We have calculated genetic diversity (Pi) from published data (Mattick et al 2021) to provided comparable data from field and or laboratory populations for other helminth species. This was done with assistance from Julie Dunning Hotopp – she is now included on the author list in the revised paper

We think the revised manuscript is considerably strengthened. Our full responses to the reviewer’s comments are shown below in red text.

**Rev 1**

**PLoS Pathogens D 24 02401**

This is an interesting ms. that describes genome sequence analysis of lab lines of the trematode *Schistosoma mansoni*, and compares them to already available genome data for wild parasites. The authors show that the lab lines, as one might expect, had less genetic variation than the wild parasites, though that the lab lines were not fully inbred. The authors discuss the utility of the lab lines in schistosome research, and present caveats to this. Overall the work is thorough, and the conclusions are justified by the data that are presented.

I think that the ms. could do quite a bit better in reaching out to a wider readership. As it stands it is readily comprehensible to those working on trematodes, but some matters wouldn’t be clear to other parasitologists, and certainly not to other pathogen biologists. I offer some suggestions in my more detailed comments below.

Thank you for this suggestion. To emphasize the relevance to other helminth species, we have added an additional sentence to the first paragraph (3^rd^ sentence) of the introduction:

“In contrast, most helminth parasites (e.g. *Trichuris*, *Heligosomoides*, filarial nematodes) used in biomedical research are obligately sexual so must be maintained in the laboratory as recombining populations. Our laboratory focuses on the blood fluke, *Schistosoma mansoni*, which have separate…. ”

Throughout, some greater care and precision is warranted, again which I also lay out below.

I’m unclear about the 19 to 24 parasites sequenced from each laboratory line (line 23; lines 119 – 121; the methods; Figure 2 and its legend). Methodologically (from line 247) was each snail infected with a single miracidium? If so, for infected snails, was it a pool (size 19-24) of the cercariae from each infected snail that was then sequenced? (As an aside, in Figure 2’s top panel is the beaker meant to show eggs in water that are hatching to release miracidia?) If it is pools of cercariae from single-miracidia-infected snails that were sequenced, isn’t this effectively a severe bottlenecking, and does this lead to the low estimates of genetic variation present with each lab line? To put it another way, if offspring of different parasites within each laboratory line were sequenced, would the estimates of genetic variation be higher? Alternatively, it is cercarial pools of single-miracidia-infected snails that were harvested, and then 19-24 of these pools per lab line that were sequenced? As it stands, the text and figure isn’t sufficiently clear to understand this rather important point.

We have now removed the figure 2 and clarified the text. In brief, we sequenced 19-24 independent parasite genotypes from each population. To do this, we infected multiple snails each with a single miracidia. Each of the parasites sequenced from a population was derived from a snail infected with a single miracidia. This ensures that each of the parasite genotypes sequenced is an independent recombinant parasite. We have added additional sentences in the materials and methods to explain this (lines 422-423).

I am surprised by the admixture results (lines 137-139) given that the lab lines are maintained with sexual reproduction and that there are decent levels of genetic variation within the lines. It makes sense that there is no mating among the lab lines (though line 371 talks about cross-lab line contamination and, I assume, crossing), but have the authors examined the admixture of each lab line separately?

Thanks for this comment. We did not conduct admixture analyses for each individual population. However, admixture groups individuals into populations based on differences in allele frequencies, and by identifying groups of individuals genotypes that meet Hardy-Weinberg expectations, and by using Linkage disequilibrium between markers. Hence the admixture results are consistent with each population consisting of recombining populations.

To my taste the Discussion is overly long and digressive, though I think this about almost all papers I review, and of many papers I read – so this might say something about my taste.

We were unable to significantly shorten the discussion. However, we hope that the subtitles allow readers to easily navigate to sections of interest

**Detailed comments**

Line is 20 to 21. “Schistosomes” and “rodents” – can all schistosome be maintained in rodents? I presume this hasn’t been tested for all species.

Mice or hamsters are typically used for laboratory maintenance of schistosomes. For this study we used Syrian hamsters. This is now detailed in the methods section (line 427). We used “rodents” in the abstract due to word limits.

Line 29. “Indicative” – this is ambiguous because given the text before this word it could refer to “all laboratory populations” or “SmBRE”.

We have now put “except SmBRE” in parentheses to make it clear that this sentence refers to “all laboratory populations (except smBRE)”(line 30)

Line 44. This “clonal” doesn’t make sense to me here. This is also repeated on line 67.

We have now replaced “*clonal*” with “*asexual*”

Lines 48 – 50. This sentence is an opportunity for the authors to give a broader perspective about *S. mansoni*, for example telling the wider leadership that this is a macroparasite, a trematode, a platyhelminth *etc*.

We have inserted a short sentence into the Author summary (ln 49-50) to state: “The blood fluke *Schistosoma mansoni* (Phylum: Platyhelminthes; Class: Trematoda) is commonly used laboratory helminth.*”*

Line 51. I don’t think that the authors can conclude that the genetic variation was ”reduced”, because they don’t present data showing the genetic variation in the ancestors of these lab parasites. Rather the authors can only say that the variation currently present in the lab lines is approximately half of that present in present day wild populations. This is repeated on line 113, line144 (here how about “significantly lower” rather than “significant reduction”?), and line 212.

Thanks for this comment. We have now reworded all of these statements. Other reviewers also commented on this and we have reworded several manuscript sections to reflect this.

Line 64. What is meant by “recombining”? Do the authors mean meiotic recombination or do they mean quasi-random sexual meeting among adult parasites, or something else?

We mean meiotic recombination. We have now stated “*where adult males and females pair, meiosis and recombination occur and eggs are produced*” in this sentence to clarify (line 76)

Line 65. The sentence beginning “successful” seems quite out of context and I suggest that it is removed or revised by the authors. As toa revision, I think the text needs to explain the reason for telling the reader this.

We have removed the word “successful” as suggested. The lack of effective cryopreservation methods for schistosomes means that continuous propagation of laboratory lines is necessary, which has important consequences for genetic variation. We have now added the sentence: “Cryopreservation of many viral, bacterial or protozoan pathogens simplifies research on these pathogens. However, while…” (lines 71-72)

Line 66. Sentence starting “Schistosomes”, could be an opportunity to explain the life cycle and particularly the genetics of the life-cycle to a wider readership. As it currently stands this sentence doesn’t make the important point that the adult parasites undergo sexual reproduction. I’ve commented above about the use of the word “clonal” and wonder if “asexual” may be better here.

We have added the phrase “, and where meiosis and recombination occur.” at the end of the sentence on line 76.

Line 76. Are these five generations sexual generations or asexual generations?

We have reworded this as “five complete parasite generations” to emphasize that this includes both sexual stages in the vertebrate host and asexual stages of the lifecycle in the snail host (lines 83)

Line 93. How about “difficulties” or “imperfect genome assemblies” rather than the (overused and often largely meaningless) “issues”?

Now changed as suggested

Line 95. Insert “assembly” after “genome” – the *S .mansoni* genome is perfect, it is just our knowledge and our assembly of it that is imperfect.

Changed as suggested

Line 96. Another “issue”, but it isn’t clear what “this” refers to.

Sentence now clarified

Line 97. This would be a place where the authors could give some brief details about the genome assembly, which otherwise is barely mentioned or metrics about it given. Also, while reading this ms. I realise I have no idea of the size of the *S. mansoni* genome assembly.

We have now included details of the current genome assembly (lines 104-107)

Line 99. “Cercariae” will be almost meaningless to non-schistosome biologists and certainly to non-parasitologists, and so this is another example of where the authors could reach out to a wider audience.

This is now rephrased to read “*Davies et al. isolated parasites that shed low or high numbers of schistosome larvae (cercariae) from the snail host from the SmPR population*” (lines 108-109) to make this work clearer and more accessible for non-schistosome biologists

Line 105. How about “the present study” rather than “this study”?

Now changed as suggested

Line 108. Figure 1 doesn’t obviously support the two alternatives presented in the text here.

We have now removed figure 1 – Review 2 also did not find this useful.

Line 119 and Figure 2. I am unclear what the 19-24 parasites per lab line are, as I explain above.

We have now removed figure 2 – Review 2 also did not find this useful. However, we have also rewritten the methods (lines 423-424) and results (lines 129-30) to clarify that we sequenced 19-24 independent parasite genotypes per population.

Lines 124 –125. What are the n’s for each of the countries?

N shows the number of independent parasite genotypes sequenced from each population.

Table 1. What is a “sample” in the heading of the second column. And, instead of “All variants” how about “Total variants”?

We have replaced “Number of samples” to “No. Parasite genotypes”, and “All Variants” to “Total Variants”

Lines 134 to 136. This is wrong in two ways. First, the four lines are separated by the vertical access, they are clustered by the horizontal axis. Two, they are separated / clustered by the principal components not the axis *per se* – a pedantic point but principal components 1 and 2 could equally well be plotted on the y and x axis, respectively.

We have rephrased as suggested (lines 147-150)

Line 153. I don’t know what this Wilcoxon test is testing and I can’t see that this is explained in the methods.

We used the Wilcoxon test to compare values from field and lab populations. This is now explained in the legend

Line 159. I don’t understand what this statistic is telling us about the ECDF – is it an analysis of the slope or the intercept, or something else? I can’t see that this is explained in the methods. Also the P value here is different than that on Figure 5B, but I think that they should be the same.

We have used an alternative permutation approach to determine whether ECDF plots are different in laboratory and field derived schistosome populations in the revised manuscript. This is now outlined in the methods (lines 567-572) and results (192-197) and in supplementary fig S4.

Line 188. Why the harmonic mean?

The harmonic mean is used rather than the arithmetic mean, because small bottlenecks have a disproportionate impact on Ne (<https://pmc.ncbi.nlm.nih.gov/articles/PMC1449138/>). This citation is now added (line 225)

Line 189. How was this number of genotypes estimated? Line 451 *et seq* of the Methods seems to use the number of miracidia but these aren’t the definitive host infective stage and how do the authors get from miracidia number to genotype number?

We estimated the numbers of parasite genotypes present in infected snails. We cannot count this directly, but we can count how what proportion of snails are infected, and estimate (from poisson distribution) the number of snails carrying 1,2,3,4….10 different parasite genotypes. We have modified the text (*lines 543-546*) in the material and methods to clarify

Table 3. Six of these columns repeat data in Figure 7. There is no legend to the Table.

The table further examines the relationship between Nc and Ne, so we feel that this duplication is justified. We have now provided a legend to better explain the table contents

Line 198. Is “N” the number of worms, and if so the number of which life cycle stage?

N is the number of adult worm genotypes. This is now specified in the text (lines 235-236)

Line 239. Sentence beginning “We observed…” doesn’t sit easily with me. Didn’t the authors observe a positive Tajima’s D, which is because of the comparative loss of rare alleles. In itself, the authors didn’t observe a loss of rare alleles (thought there is Figure 5B, though there are no analyses of these date). [I downloaded what purported to be Figure S1 (9 histograms with the axes labelled ‘Number of sites” and “Sample size’, but this isn’t obviously what line 156 refers to.] Notwithstanding, my comment concerns the logic of what is observed and what is inferred, which I’m not sure is correct in this sentence.

Thanks for pointing this out. To improve this section, and remove the inferences, we have changed “(i) Loss of rare alleles” to “lower numbers of rare alleles” and (ii) Reduction in LD decay to “Differences in LD decay.

Line 241. Insert “comparative” before “deficit”?

Done

Line 247. Why “reduced … recombination events”?; and see my comment on line 64.

This is now changed to “meiotic recombination” events (line 289)

Line 262. What are the “groups”?

We have removed “among all groups” – this is not needed in the sentence

Line 264. The sentence beginning “A microsatellite” seems out of context. Explain the reason for telling the reader this.

We have removed microsatellite-based from this sentence.

Line 299. ExamineS.

Now modified

Line 306. I wonder whether “association” is helpful here since a GWAS seeks statistical associations between the phenotypic trait and genetic markers, but GWAS works because genetically there is linkage (another type of association) between a locus underlying trait and genetic markers.

Our preference is to retain association in this sentence, because this is the basis of Genome wide association methodologies. Linkage would be appropriate for analysis of genetic crosses and linkage analyses.

Line 347. “so are typically…” was this exclusion done here? If so, I didn’t get that from reading the methods.

Highly variable genes are poorly genotyped using illumina sequencing due to from poor mapping of reads in these regions. We have rephrased this section to read “poorly genotyped” (line 403) rather than “typically excluded”, to show that “exclusion” is not manual, but occurs due to the read mapping process.

Line 374. Perhaps clarify that the heads were what was sequenced.

We have modified the text to *“…..and extracted gDNA for sequencing……” (line 437-438)*

Line 464. How were the 95% confidence intervals calculated?

This is detailed in the packages used to generate the estimates – we have provided the citations for interested readers (line 547 and 549).

Figure 2A. Why are the sex chromosomes not shown?

I believe that you mean Fig 4A in the original submission. We focused on autosomes, and did not include sex chromosomes, because the number of males (ZZ) and females (ZW) differed between populations, so could result in biased measures of genetic diversity. However, all the sequence data are available, so interested readers can further analyze these data.

Figure 6A. This plot shows dots through which lines are drawn. What are the dots and what are the lines? The Methods doesn’t obviously explain this.

The dots show levels of LD between pairs of markers situated on the same chromosome and separated by the distance (shown on the x axis). The lines are fitted to the dots to examine the relationship between physical distance and LD, and that rate of decay in this parameter. We have now explained this more clearly in the figure legend (lines 880-881).

Figure 8. The y-axis label is unclear to me, and I can’t see that “H0” is explained in the Figure legend or the text,

We have modified the 3^rd^ sentence of the figure legend to read: “*H_0_ Indicates Heterozygosity at time 0” (line 891)*

**Rev 2 (steve Doyle)**

2024_PLoS_Pathogens_Schisto_lab_strain_diversity

Jutzeler and colleagues have analysed the genetic diversity of five laboratory-adapted populations of *Schistosoma mansoni* using whole-genome sequencing. Surprisingly, they found that, despite being isolated for decades and hundreds of generations, the laboratory-adapted isolates were still relatively genetically diverse compared to field isolates. These data have important implications for understanding genetic and phenotypic variation in laboratory models, which are usually considered to not be diverse. The discussion was particularly good in highlighting the strengths and limitations of having such diversity in lab populations and the fact that it might be exploited to understand diversity and trait variation better, especially if single-worm phenotyping is used. Similarly, extending this to other helminths is important, although there are more examples that could have been drawn from.

Overall, the manuscript is well written, its analyses robust, and its conclusions are generally well supported. Below are some questions and comments that I think are worth considering. Addressing them may strengthen the manuscript and clarify potential uncertainties for the reader.

Kind regards,

Stephen Doyle

Wellcome Sanger Institute

General comment

- An overall theme is comparing lab to field strain diversity; however, one aspect of this analysis is the data have not been generated in the same way, i.e., whole genome sequencing was used for the lab stains, and exome sequence was used for the field strains. Filtering variants to include only coding variants helps bring these two approaches together. However, are there other sources of variation that need to be accounted for? For example, how concordant are WGS and exome capture variants? Are there coding sequences that the exome capture does not target, and do you account for this here? Is there any allelic imbalance caused by the exome capture that might not be affected by WGS? This technical variation could use some recognition in the discussion.

Thanks for raising these points.

- **how concordant are WGS and exome capture variants? Are there coding sequences that the exome capture does not target, and do you account for this here?** In the initial manuscript, we filtered the whole genome sequence and exome capture datasets by only to generate the dataset for comparison of variation by only including coding regions. In retrospect, this was suboptimal and can potentially result in some SNPs being scored in only exome capture data, or only in WG sequence data. To improve comparability, we have now applied filters within each population and score variants that are scored in at >80% samples in each of the lab and field collected populations examined. This reduces the total number of variants, but improves the rigor of comparisons between WGS and exome datasets.
- Is there any allelic imbalance caused by the exome capture that might not be affected by WGS? Exome capture can result in allele specific capture in genome regions showing high levels of divergence. However, exome capture is extremely effective for sequencing alleles showing up to 10% divergence (https://pmc.ncbi.nlm.nih.gov/articles/PMC3472323/). Furthermore, bias due to allele specific capture is minimized because exons are typically more conserved than non-coding regions. We therefore don’t believe that this will be an important source of bias for this dataset. We have now discussed potential bias due to use of capture based methods in the discussion (line 409-413).
- Reading throughout, I keep returning to the statements of “49%... diversity retained” and “51% reduction.” You have multiple independent populations of both field and strain, within which there is distinct variation within each, so it doesn't make sense to me to make a singular statement like this. “Retained” and “reduction” suggested you measured diversity in the starting population, time passed, and then measured again, i.e., one population changed over time. However, with independent populations, this statement can’t be made, at least not precisely, as there is no defined history or genetic relationship between field and lab strains. Moreover, some of the variations in the lab populations will be new since they were isolated and, therefore, distinct, i.e., different between the same lab isolates kept in different labs, for example. I think this deserves some thought as to how you describe it. Perhaps framing it as the “relative levels of genetic diversity” might be suitable, and simply that there is more variation in lab strains than expected.

Rev 1 made the same point. We have now changed the language using in the abstract, results and discussion to make this clear and explicitly discuss “relative levels of genetic diversity”.

Note that with the new analysis using a more rigorously filtered dataset, laboratory samples have 62% less variation relative to field collected samples

- I don't think Figure 1 or Figure 2 are meaningful or detailed enough to warrant being in the main text figures.

We have now removed figures 1 and 2. We agree that these are not informative.

Specific comments

Line 96: “This approach contributed to a much-improved genome “

- This seems to end prematurely, as there is an even more contiguous genome assembly (*S. mansoni* V10 in WBPS; Buddenborg et al bioRxiv) that was also improved with DNA derived from single miracidium infections.

We have now added an addition sentence: “The current version 10 of the *S. mansoni* genome assembly was further improved using libraries derived from single miracidia, and encodes seven chromosomes, ZW sex chromosomes, and is ~ 391 Mb in size (ref).” (lines 104-107)

Line 123: “Detailed information about these variants is listed in Table 1.”

- Given the sample sizes, the minor allele frequency filter may or may not retain SNPs present only in a single individual. E.g. Niger will be very susceptible to this with only nine individuals.

Filtering for variants present at >0.05 frequency retains SNPs present in a single individual in the case of Niger. This is why total SNP numbers and MAF filtered SNP numbers are very similar in Niger. However, for other populations the lowest sample size is 19 (38 genomes). Hence, MAF filtering will retain variants present in at least two alleles, and are likely to be reliable. We have now included a footnote in Fig 2 to explain this.

- How meaningful are these rare variants, and are they found in multiple populations to give some confidence that they are real?

We have used MAF filtered variants for all comparative analyses between field and laboratory populations, to improve confidence in variant calls. As noted in comment above, MAF filtering will result in use of variants present in ≥2 copies in all populations except Niger. We did not determine whether rare alleles were detected in ≥2 populations in this study.

- Did you look at the number of SNPs fixed in different populations?

We did not, but this is an excellent idea. Increased drift in small populations is expected to result in increased fixation of SNPs. Furthermore, reduced efficiency of selection may result in fixation of mildly deleterious alleles. We have now added an extra panel in Figure 1 (Fig 1C), which shows the number of variants that are fixed in each of the lab populations. We specifically measured the numbers of derived variants that reached fixation using an outgroup (*S. rodhaini*). We observed more fixed derived variants in laboratory populations than field populations. Furthermore, an especially large number of fixed SNPs were fixed in smBRE laboratory populations. This is consistent with bottlenecks/low Ne in this population. These results are now reported in Figure 2C, detailed in the methods (lines 506-512), results (lines 174-178) and discussion (lines 283-286).

Line 132: “Principal component analysis (PCA) and admixture”

- This is clear that the laboratory populations are distinct, which is great. Perhaps not in the same way, though; for example, there is minimal variation in PC1 separating four strains. Do you have any idea what is driving the divergence on PC1?

We suspect that the elevated levels of fixed SNPs (see comments above) in smBRE drive PC1. We have not further examined this this hypothesis to avoid unnecessarily complicating the manuscript.

- However, it seems like a missed opportunity that you have not compared them to the field populations. I suggest that if not here, then in the supplement, you should show all of the samples in the PCA.

We have now generated a PCA plot from exon data only, that includes field and laboratory populations. This is shown in the supplementary figure S1

- Given you have both a lab and field strain from Brazil, it would be interesting to know how similar they were.

We actually have two lab populations (smLE and smBRE) and one field population (Brazil) from Brazil. SmLE and Brazil cluster together in the PCA plot (supplementary figure S1). However, smBRE is quite distant on the PCA plot. We suspect this results from elevated numbers of fixed alleles and low diversity in smBRE.

Line 141: “Nucleotide diversity in S. mansoni laboratory and field populations”

- It is interesting that SNP variation is relatively consistent across the genome and that it is not just restricted to chromosome ends, which is commonly seen. It might suggest there are technical aspects of variant calling in these regions or true hotspots.

To further examine patterns of variation across the chromosome, we examined pi in 25, 5 or 2kb windows across the genome. There is no increase in mean levels of SNPs at the chromosome ends with these different window sizes. However, there is an increase in variance in SNP number. This analysis is shown in the supplement Fig S2, and we include a sentence to describe these patterns in the text (lines 162-164).

- It would be helpful to show some pairwise comparisons of nucleotide diversity using Dxy or something similar. Even a ratio of Pi would work. It would help highlight population-specific regions of high diversity.

We have now included plots of average number of nucleotide differences per site (D_XY)_ for pairwise comparisons of populations to identify genome regions showing large differences in allele frequency between populations (Figure S3). This figure includes a list showing the gene content of genome regions with elevated D_XY_. We have described the D_XY_ results in lines 180-185.

Line 143: “Statistical analysis using a Kolmogorov-Smirnov test showed a significant reduction in diversity (51%) in laboratory populations (D = 0.403, p < 0.001).”

- This is a little confusing, as it is unclear what is being compared from the figure.

First, the KS test compares whether two datasets are drawn from the same distribution—so are you comparing all fields against all labs as two groups? If yes, is that appropriate?

Reviewer 1 also raised this point. The test we use did compare comparing all field against all lab samples as two groups. We agree this is not ideal. We have used an alternative permutation approach to determine whether ECDF plots are different in laboratory and field derived schistosome populations in the revised manuscript. This is now outlined in the methods (lines 567-572) and in supplementary fig S4.

- The 51% reduction is based on what exactly? Cumulative diversity in lab vs field? The figure doesn't show this, given that you have shown each population.

We measured the mean Pi in lab populations (by calculating pi for each lab population, then taking the average) and comparing to the mean Pi in Field populations. This is now stated in the methods (lines 502-505). Note that with the more rigorously filtered dataset, we now observe 62% less variation in laboratory parasite populations.

Line 153: “Wilcoxon test”

- What are you testing here, exactly?
- The test is not significant; however, your description of the test does not reveal that. For example, you say that “all field populations showed negative values ” with a non-significant test. Does that mean that statement is not supported? It's best to clarify.

We used Wilcoxons test to compare values observed in field and laboratory populations. This is now stated in the legend.

We have removed the first sentence of the paragraph (“*Tajima’s D revealed a stark contrast between laboratory-maintained and field populations*”) which suggests that the result is significant.

Line 158: “significant differences between allele frequency spectra for laboratory and field populations”

- Again, it needs to be clarified what the KS test is comparing.

We have used an alternative permutation approach to determine whether ECDF plots are different in laboratory and field derived schistosome populations in the revised manuscript. This is now outlined in the methods (lines 567-572) and in supplementary fig S4.

Line 166: “To compare LD decay curves, we measured the distance at which LD is reduced to r2 = 0.5 (LD0.5, Figure 6B).”

- The problem with this is none of the populations get to 1, and that Tanzania doesn't get to 0.5, so it has no value.
- It would be better to calculate the LD at 50% of the max LD per population
- It should also take into account the background LD, which could be determined by the LD between variants from different chromosomes.

We respectfully disagree on the first two points. LD is expected to converge on 1 as sites become closer. That LD starts at <1 for all populations, reflects the fact that we have plotted the mean LD between sites up to 1kb apart. For this reason, LD50 is calculated as 50% of the theoretical maximum in other papers using this approach. (e.g. Reich et al 2001, https://pubmed.ncbi.nlm.nih.gov/11346797/). We have now included this citation in the revised paper

Regarding the third point – this is an excellent idea! The high LD seen in Niger between sites up to a million bp distant, for example, is most likely explained by sample size. We have added a plot showing background LD between unlinked sites (marker pairs on different chromosomes) to address this (Fig S4).

Line 167: “Figure 6B”

- This is the most telling difference between lab and field populations, and given the WGS vs. exome confounder, the difference is likely underestimated.

We agree. We have also compared LD across populations using the exon data only in Fig S5

Line 169: “LD decay was nearly significantly”

- It can't be nearly significant. It either is, or it isn't. Here, it is not significant. Please change.

Thanks for pointing this out – now changed!

Line 196: “Simulations of genomic diversity in populations of different size”

- This is neat.
- How did you account for the lifecycle in your simulation? The methods are not very clear and do not account for an intermediate host or clonal stage in the life cycle.

The simulations do not account for features of the schistosome lifecycle. Instead they are generic simulations that examine the loss of heterozygosity in recombining diploid organisms maintained at different census sizes. Our aim was simply to evaluated expectations given different sized laboratory populations. We have added text to the methods to clarify this (lines 560-562).

- If I interpret this correctly, it suggests that with a comparable effective population size and generation time, one might not expect to lose much diversity. Is that interpretation correct? If so, the overall result of “retaining” diversity is not surprising.

This is correct. These simulations are broadly consistent with observed levels of variation in laboratory samples, but only if the number of adult parasites founding each subsequent generation is quite large. We keep records of the number of snails shedding cercariae that are used to infect rodents during laboratory maintenance. This is a proxy for the numbers of adult worm genotypes present in rodent hosts. Hence, we can estimate the census size in each generation in the laboratory maintenance. A caveat here is that we do not know the methods used for laboratory maintenance of these parasite lines prior to establishment in our laboratory. Importantly, these simulations are intended to show how much variation is expected to be maintained in parasite populations of different size.

- How dependent is this result of maintaining diversity on the actual sample in which diversity is maintained? Could you get this result independently (including considering the life cycle)?

These simulations examine the loss of variation, rather than absolute levels of variation expected. These simulations are also deliberately simple, and generally applicable to all sexually recombining populations. We have included an additional sentence explaining these simulations in the methods (lines 560-562)

Line 216: (Table 4)

- I’m not convinced a table like this enables a meaningful comparison between studies or organisms. There is so much variation between all of these examples - time, population size, bottlenecks, life cycles etc - and presenting a table with individual figures and a reduction in diversity almost implies this is a biological truth to these numbers rather than a random collection of anecdotal evidence.
- Nonetheless, I am more concerned that you have simplified your data into a single data points, when clearly the rest of your data shows variation between samples and between groups.
- I suggest you remove the table. It is OK to reinforce that “domestication” or “lab adaption” generally results in loss of diversity. Still, I think it is best not to make comparisons when they can’t properly be compared.

We respectfully disagree and would like to keep this table, but as a supplement (Table S2). The table lists similar studies that compare genetic diversity in domesticated or laboratory adapted organisms with that in field collected populations. Where several lab and field populations have been compared, we report average values from lab and field. The table makes the point that the difference in diversity observed between field and lab schistosome populations falls in the spectrum of that observed in studies of other domesticated organisms. In particular, we think that the table is useful to show parasitologists that there is a large literature describing comparable data from free living organisms.

Line 221: “laboratory schistosome populations are typically founded by collecting eggs from one or more patients, each of which may be infected with hundreds of adult worms”

- This is hypothetical—I appreciate we cannot know exactly—but let's assume this is true. Given your effective population size estimates, it would potentially suggest there is effectively no loss of diversity from a small founder population over hundreds of generations. Does this make sense?

We included the simulation to examine how adult census size might be expected to impact the loss of variation from laboratory populations. These simulations suggest that substantial amounts of variation may be retained with 100-200 breeding adults each generation, even after 400 generations in the laboratory. We have added a sentence to the discussion to better explain the conclusions from these simple simulations (lines 271-273)

- I think this needs some more thought and discussion. Comparison to the field populations is actually too broad, and would be more informative to compare to the founder population.

We certainly agree that comparison to the founder population would be ideal, but unfortunately we cannot make this direct comparison. The caveats section of the discussion now lists this weakness (lines 399-402).

Line 332: Relevance to other helminths

- This is a really important point to raise, and might prompt similar studies to better understand variation in other laboratory models.
- However, you briefly describe only one helminth and C. elegans - what about the others? There are a few I can think of, eg. Brugia malayi (Mattick et al 2021 PLoS NTD).
- Given you have made a point to illustrate it, I think it is worth fleshing this out a little more.

Thanks for bringing the *Brugia* reference to our attention. We have now added this citation and strengthened this section. The Mattick et al paper contains an informative comparison of sequence variation in *B. pahangi* maintained in the lab since the 1970s with adult worms collected from a Malaysian cat. Similarly, this paper documents genetic variation in found B. malayi lab populations maintained in the laboratory since the 1960. The corresponding author on this paper (Julie Dunning-Hottop) generated allsites VCF files from which we have calculated Pi for these populations and included a new summary table (Table 4). Julie Dunning-Hottop is now included as an author on the resubmission. We have also now included *Mattick et al 2021* data on *B malayi and pahangi* to the discussion (lines 385-393).

Line 414: “we lifted the file over to v10 of the S. mansoni reference genome (Wellcome Sanger Institute, project PRJEA36577) using LiftoverVcf.”

- How accurate is this? What validation did you do?
- Why did you not simply remap and call variants again?
- To what degree might this influence your results?

We did this for two reasons:

First, V9 and V10 are extremely similar, so do not justify the time needed to realign >200 sequences. Second, based on the chain file, minimal numbers of variants failed to carry over

Line 420: “and > 56% missingness,”

- This is an interesting number - how did you come up with it?
- There is also a difference in the filtering strategy between the lab and field samples - what effect might this have on the comparison?

**The initial draft simply compared SNPs scored in the exome from NGS data (lab samples) or exon capture samples (field samples), resulting in 56% missingness. In retrospect, this was not an ideal approach. In the revised manuscript we have now filtered data to include only variant sites that were genotyped in >80% of samples in every population examined. This reduces the numbers of variant scored but results in more comparable scoring of variation among WGS and exome capture samples. The revised filtering approach is now detailed in the methods (lines 489-490)**

**Rev 3**

**Reviewers' Comments:**

Reviewer's Responses to Questions

**Part I - Summary**
Please use this section to discuss strengths/weaknesses of study, novelty/significance, general execution and scholarship.

Reviewer #1: See attached

Reviewer #2: See attachment

Reviewer #3: The manuscript by Jutzeler and colleagues describes their discovery of high levels of genetic diversity in schistosome populations maintained in the laboratory. This is an important and relevant finding, with bearing on the use of laboratory helminth populations to study the genetic basis of traits such as drug resistance. The experimental design seems robust, the genomic analyses and simulations seem appropriate, and the data support the claims. The paper is clearly written and was enjoyable to read, although I felt in a couple of places there was an expectation that the reader was already familiar with the papers cited; please always state the key point briefly rather than simply providing a reference for the reader to look up. I have no major corrections but have several minor edits or comments/queries.

Thanks for your enthusiastic summary. We have now stated the key points of papers sited in several sections of the manuscript

**Part II – Major Issues: Key Experiments Required for Acceptance**
Please use this section to detail the key new experiments or modifications of existing experiments that should be absolutely required to validate study conclusions.

Generally, there should be no more than 3 such required experiments or major modifications for a "Major Revision" recommendation. If more than 3 experiments are necessary to validate the study conclusions, then you are encouraged to recommend "Reject".

Reviewer #1: See attached

Reviewer #2: (No Response)

Reviewer #3: None

**Part III – Minor Issues: Editorial and Data Presentation Modifications**
Please use this section for editorial suggestions as well as relatively minor modifications of existing data that would enhance clarity.

Reviewer #1: See attached

Reviewer #2: (No Response)

Reviewer #3: 72. Please state the country the SmNMRI population was isolated from.

SmNMRI originated in Puerto Rico – this information is now included with citation (line 78)

Fig 1. Panel on RHS would be improved by showing males and females within each pair in different colours.

We have now removed this figure following suggestion of Rev 1 and 2

Fig 3. Did you also generate a PCA with the exonic data only to allow inclusion of field samples?
Rev 2 also suggested this. We have now included this plot in the supplementary data (Figure S1).

144. The 51% reduction presumably reflects the very high diversity of the Tanzanian field population (and the low diversity of SmBRE), but as far as is stated in the paper, none of the lab isolates originated from Tanzania. What is the reduction if you do the most relevant pairwise comparisons e.g. each Brazilian lab isolate with the Brazilian field sample?

We have now added a sentence to compare diversity between lab and field populations after exclusion of the diverse Tanzanian population to address this (lines 169-170). We have two laboratory populations from Brazil: smBRE and smLE.), these populations show 85% and 54% less variation than the Brazilian field population (Brazil). This is now addressed in lines 171-173.

148. Did you look at which genes were encoded in regions of high nucleotide diversity across the different isolates? In Discussion, suggest that balancing selection due to host immune response could maintain genetic variation as per Stevens et al 2023, so might expect to see enrichment for gene families that interact with the host immune response in these loci?

Reviewer 2 also suggested examining this. We have now included plots of average number of nucleotide differences per site (D_XY)_ for pairwise comparisons of populations to identify genome regions showing large differences in allele frequency between populations (Figure S3). We have also included a table showing genes with elevated listing the gene content of genome regions with elevated D_XY_.

169. I don’t think ‘nearly significantly’ is appropriate description.

Thanks for pointing this our – now corrected

193. ‘The reasons for this are explained elsewhere [19].’ Please state the reasons briefly.

This is explained in the methods section (line 435-436) and resulted from a laboratory contamination event with LE parasites. We have now replaced “The reasons for this are explained elsewhere” with “as a result of a laboratory contamination event”. For this reason we used archived adult smBRE worms, collected prior to contamination for this project.

199. Are overlapping generations relevant for laboratory maintenance of schistosome populations?

We have now removed the showing reduction of variation expected with overlapping generations simulations from Figure 6. We have also updated the figure legend and associated text sections accordingly. We agree that this is an unnecessary complication.

256. Worth also commenting on the distinct allele frequency spectra here (Fig S1).

We have modified this sentence to read “…., *SmBRE had a strongly negative Tajima’s D and site frequency spectra like the field collected populations*.” (lines 304-305)

Table 4. Could be interesting to include data from lab and field isolates of other well studied outbreeding helminth populations (e.g. *C. remanei* and *H. contortus*) if available for comparison.

We have now moved table 4 from the original submission into the supplementary material (Table S2 in revised manuscript). However, the revised manuscript contains a new table (table 4) describing genetic variation in *Brugia malyi* and *Brugia pahangi*. Laboratory maintained parasites of both species maintain extensive variation despite being maintained for 50-60 years in the laboratory, consistent with our findings for *S. mansoni*.

349. Please add ‘or PacBio’

Now added

373. Individual males and female heads were sequenced for SmBRE only, but previously stated (line 363) the avoidance of sampling adults in case derived from clonal cercariae from a single snail – is there a risk this did happen for SmBRE and relates to the very low genetic variation measured in this isolate? Good to highlight any technical differences in the discussion.

We measured relatedness between isolates – none of the smBRE parasites sequenced were clonally identical. We can therefore eliminate the explanation. We have now mentioned this in the discussion (lines 301-302)

375 and 384. Do you expect any bias from the WGA step that could affect the results?

We have previously examined accuracy of genotypes derived from whole genome amplified schistosome material, by examining genotypes for F1 progeny and their parents (Valentim et al. 2009) This revealed an extremely high accuracy (0.45% error rate). We have now cited this reference in the methods (line 449-451)

414. Unclear why lifted merged vcf to different genome assembly?

See answer to Reviewer 2: We did this for two reasons. First, V9 and V10 are extremely similar, so do not justify the time needed to realign >200 sequences. Second, based on the chain file, minimal numbers of variants failed to carry over

PLOS authors have the option to publish the peer review history of their article ([what does this mean?](https://links.us1.defend.egress.com/Warning?crId=675c2bc70a771b18b0ccaa8e&Domain=txbiomed.org&Lang=en&Base64Url=eNpVTMtOwzAQ_BqOG9tJE8VIXIrIIXAoIAE9bjebR5vYlm0I9OtxQUJiNCPtzszuGKML10JEj3TKuJui9RPOCxoc2GdkF3H7IMVv7SpvEo_23RucQ-ZmGzLrh-RdRodxtAObkPaL_p4Bmg4cswfPHxOv4LwlDqlTzJb-JeMU0s2XUEIq2UulC5ISO0QNikvFBddQYFXAhqgETboGmfd46KuDYiSQPxDr-a3d5UNzX7b7LT_KvDrbPT9t_Ovysm0_d_FoTvq5oZXvblStvwF3Blbn&@OriginalLink=track.editorialmanager.com)). If published, this will include your full peer review and any attached files.


If you choose “no”, your identity will remain anonymous but your review may still be made public.


**Do you want your identity to be public for this peer review?** For information about this choice, including consent withdrawal, please see our [Privacy Policy](https://links.us1.defend.egress.com/Warning?crId=675c2bc70a771b18b0ccaa8e&Domain=txbiomed.org&Lang=en&Base64Url=eNoliskOgjAUAL_GYzdQpCZe0Hgwxi2o8WQejwKNYJtSQfx6jU7mMslU3tt2xph3gHeqcu2N01A38IBSOYqmYYsNZ_9tFKy-9n1PbW1aalz5Tet0BzgQa2qNAxOMC15wIUPkHHIASYSaCBWqmIQQhWSMOCESZUx4UEBWRJlQgIT_YO5YNbd-nB_KNLXJab310_MS33e9Cy7uicM-ctULks4E17mI5QfYOD3Y&@OriginalLink=track.editorialmanager.com).

Reviewer #1: No

Reviewer #2: **Yes:** Stephen R. Doyle

Reviewer #3: No

[NOTE: If reviewer comments were submitted as an attachment file, they will be attached to this email and accessible via the submission site. Please log into your account, locate the manuscript record, and check for the action link "View Attachments". If this link does not appear, there are no attachment files.]

**Figure resubmission:**

While revising your submission, please upload your figure files to the Preflight Analysis and Conversion Engine (PACE) digital diagnostic tool, <https://pacev2.apexcovantage.com/>. PACE helps ensure that figures meet PLOS requirements. To use PACE, you must first register as a user. Registration is free. Then, login and navigate to the UPLOAD tab, where you will find detailed instructions on how to use the tool. If you encounter any issues or have any questions when using PACE, please email PLOS at [figures@plos.org](mailto:figures@plos.org). Please note that Supporting Information files do not need this step. If there are other versions of figure files still present in your submission file inventory at resubmission, please replace them with the PACE-processed versions.

**Reproducibility:**

To enhance the reproducibility of your results, we recommend that authors of applicable studies deposit laboratory protocols in protocols.io, where a protocol can be assigned its own identifier (DOI) such that it can be cited independently in the future. Additionally, PLOS ONE offers an option to publish peer-reviewed clinical study protocols. Read more information on sharing protocols at <https://plos.org/protocols?utm_medium=editorial-email&utm_source=authorletters&utm_campaign=protocols>
